# CD14^+^CD16^+^ monocyte transmigration across the blood-brain barrier is associated with HIV-NCI despite viral suppression

**DOI:** 10.1172/jci.insight.179855

**Published:** 2024-09-10

**Authors:** Veronica Veksler, Rosiris Leon-Rivera, Lazar Fleysher, Jairo Gonzalez, Johnny A. Lopez, Leah H. Rubin, Susan Morgello, Joan W. Berman

**Affiliations:** 1 Department of Pathology, Albert Einstein College of Medicine, New York, New York, USA.; 2 Department of Neurology, Icahn School of Medicine at Mount Sinai, New York, New York, USA.; 3 Department of Neurology, Johns Hopkins University, Baltimore, Maryland, USA.

**Keywords:** AIDS/HIV, Cell migration/adhesion, Monocytes

## Abstract

HIV-associated neurocognitive impairment (HIV-NCI) affects 15%–50% of people with HIV (PWH), despite viral suppression with antiretroviral therapy (ART). HIV neuropathogenesis is mediated, in part, by transmigration of infected CD14^+^CD16^+^ monocytes across the blood-brain barrier (BBB) into the central nervous system (CNS). In the CNS, CD14^+^CD16^+^ monocytes contribute to infection and activation of parenchymal cells, resulting in production of neurotoxic viral and host factors that cause neuronal damage. Mechanisms by which CD14^+^CD16^+^ monocytes contribute to HIV-NCI have not been characterized in a study population of PWH on ART without contribution from confounders that affect cognition (e.g., substance use, hepatitis C virus coinfection). We assessed cognitive function, PBMC transmigration across the BBB, and neuronal health markers in a well-defined cohort of 56 PWH on ART using stringent criteria to eliminate confounding factors. We demonstrated that PWH on ART with HIV-NCI have significantly increased transmigration of their CD14^+^CD16^+^ monocytes across the BBB compared with those with normal cognition. We showed that hypertension and diabetes may be effect modifiers on the association between CD14^+^CD16^+^ monocyte transmigration and cognition. This study underscored the persistent role of CD14^+^CD16^+^ monocytes in HIV-NCI, even in PWH with viral suppression, suggesting them as potential targets for therapeutic interventions.

## Abstract 2

CD14+CD16+ monocyte transmigration across the blood brain barrier is associated with cognitive impairment in people with HIV who are virally suppressed and without significant confounders.

## Introduction

Antiretroviral therapy (ART) is successful in controlling HIV infection and prolonging the lifespan of people with HIV (PWH), transforming HIV infection from a terminal illness to a chronic disease. However, PWH on ART continue to be affected by HIV-associated comorbidities, including HIV-associated neurocognitive impairment (HIV-NCI) (1). HIV-NCI affects 15%–50% of PWH on ART, encompasses a wide spectrum of cognitive deficits that negatively influence quality of life, and is an independent risk factor for mortality (2, 3). There is no specific therapy for HIV-NCI for PWH on ART (4). Thus, it is imperative to identify potential therapeutic targets to reduce or eliminate this comorbidity.

Viral reservoirs persist in the central nervous system (CNS) despite ART (5–7). These reservoirs are seeded and reseeded, in part, by entry of infected monocytes into the brain (8–10). Once in the CNS, monocytes can release virus as well as differentiate into long-lived macrophages, spreading infection to other parenchymal cells (11, 12). Even with ART, neurotoxic viral and host factors are released that ultimately cause neuronal injury and loss, resulting in CNS dysfunction (13, 14). In the context of ART, chronic low-level inflammation leads to ongoing recruitment of additional immune cells into the CNS (15, 16). A subset of monocytes — CD14^+^CD16^+^, intermediate, or mature monocytes — is implicated in HIV neuropathogenesis. Mature CD14^+^CD16^+^ monocytes are more susceptible to HIV infection than other monocyte subsets (17). PWH on ART have an increased proportion of CD14^+^CD16^+^ monocytes, comprising as many as 40% of monocytes in circulation, compared with 5%–10% in uninfected individuals (18, 19). CD14^+^CD16^+^ monocyte transmigration into the CNS is mediated by chemokines, including the monocyte chemoattractant CCL2, which is elevated in the brains of PWH on ART (15, 16).

Several factors have been associated with HIV-NCI and cognitive decline, including ART nonadherence, substance use, and hepatitis C virus (HCV) coinfection (20–22). Studies that have characterized HIV-NCI included cohorts with different percentages of participants taking ART, with variable substance use, and/or HCV coinfection. In a previous study with participants that were both on and off ART and were heterogenous with regard to HCV coinfection and illicit substance use, we showed that cognitively impaired PWH had higher transmigration to CCL2 of their CD14^+^CD16^+^ monocytes across an in vitro model of the blood-brain barrier (BBB) than PWH with normal cognition (12). Viral load, substance use, and HCV alone are independently associated with cognitive impairment and HCV is a monocyte-tropic virus. Therefore, reported associations with HIV-NCI in our previous study, and in other studies in which substance use and HCV coinfection were not excluded, could be driven, in part, by the presence of these factors.

In the present study, we characterized the role of CD14^+^CD16^+^ monocytes in HIV-NCI in the context of viral suppression with a standard ART backbone (tenofovir-emtricitabine), while minimizing effects of confounders by excluding individuals with active substance use and HCV coinfection. Other medical comorbidities with potential for nervous system impacts were identified by participant interview and review of the medical record. We performed cognitive testing of study participants and characterized CCL2-mediated transmigration of their PBMCs using an in vitro BBB model. Proton magnetic resonance spectroscopy (^1^H-MRS) of the brain was done for all participants. With this in-depth characterization, we examined the relationship between the transmigration of CD14^+^CD16^+^ monocytes across the BBB, cognition, and neurometabolite markers of damage in the setting of efficacious ART, excluding the major confounders of HCV infection and substance use.

## Results

### Study population characteristics.

The study population was composed of 56 adults with documented HIV infection, recruited on the basis of being stably on an antiretroviral regimen that included tenofovir and emtricitabine for a minimum of 3 months, whose last plasma viral load derived from clinical care was undetectable. Potential participants were excluded if they had histories of coinfection with HCV or active substance misuse, confirmed on study by negative HCV serology and urine toxicology for illicit substances. Demographic and HIV characteristics of study participants are shown in Table 1. On the cognitive assessment, 32 (57%) had HIV-NCI. All study participants reported adherence to their ART regimens, and 69% had plasma HIV loads that were under 20 copies/mL or were undetectable, with the remainder having fewer than 1,000 copies/mL. We compared relevant demographic and HIV characteristics between those with HIV-NCI and those with normal cognition and found no significant differences (*P* < 0.05, 2-tailed *t* test, Wilcoxon’s rank sum test, or Fisher’s exact test) (Table 1).

### CD14^+^CD16^+^ monocytes are increased after transmigration across the BBB in response to CCL2.

**Figure 1:**
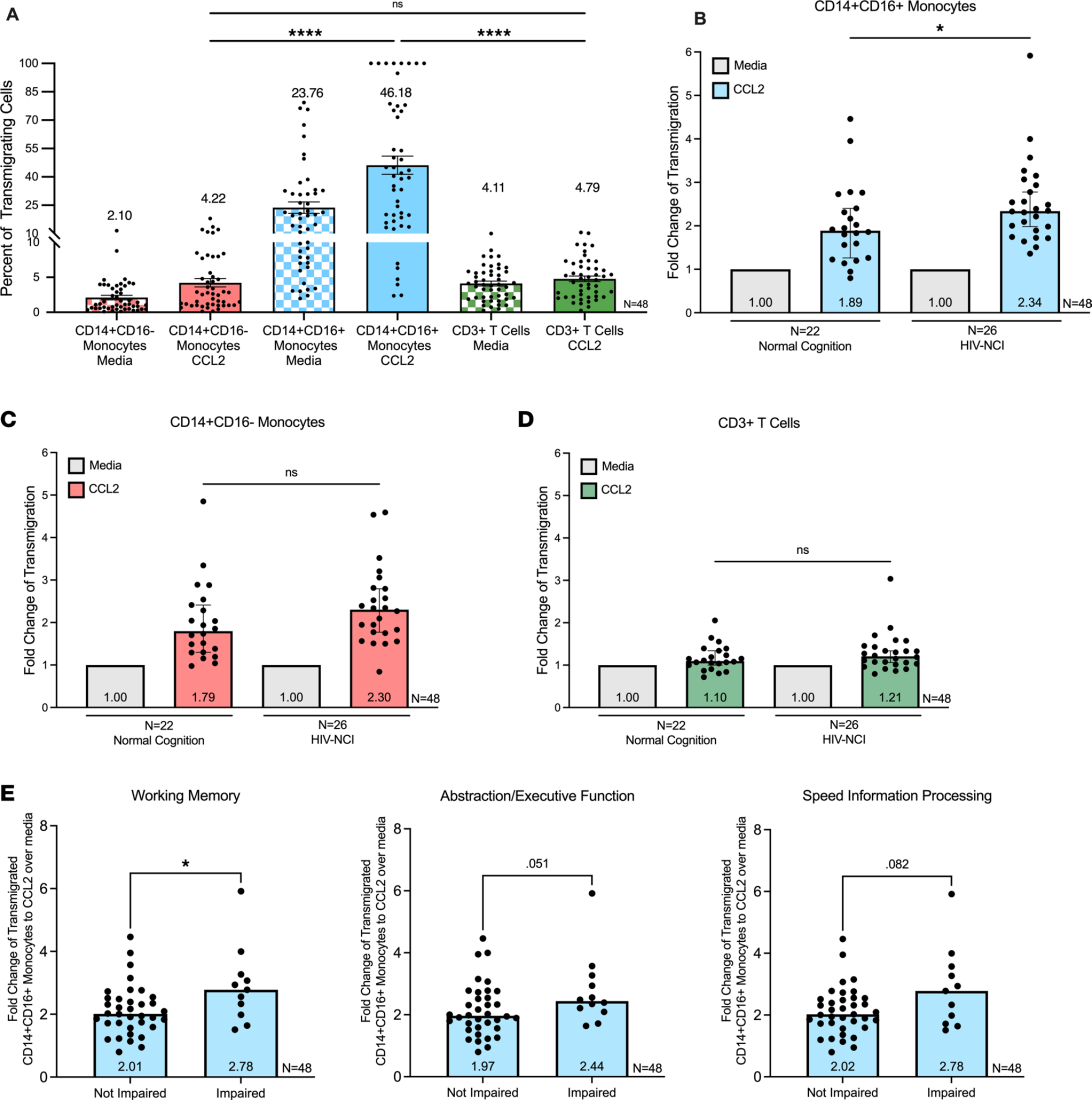
CD14^+^CD16^+^ monocyte transmigration across the BBB is higher for PWH on ART with HIV-NCI than for those with normal cognition. (**A**) The percentage of transmigration for each leukocyte subset across the BBB was calculated based on its proportion in the total cell population added to the coculture. Data are represented as mean ± SEM. Significance was determined using ANOVA with Tukey’s multiple-comparison test; ****P < 0.0001. To compare transmigration between participants, a fold-change in transmigration to CCL2 of each leukocyte subset was set relative to transmigration to media with diluent, set to 1. Fold-changes of transmigrated CD14^+^CD16^+^ monocytes (**B**), CD14^+^CD16^–^ monocytes (**C**), and CD3^+^ T cells (**D**) were compared between PWH on ART with HIV-NCI or with normal cognition (**B**–**D**). (**E**) Transmigration of CD14^+^CD16^+^ monocytes was compared between participants with and without an impairment in individual cognitive domains of working memory, abstraction/executive function, and speed of information processing, regardless of overall cognitive status. *n* = 48; data represented as medians ± 95% CI. Significance was determined by Wilcoxon’s rank sum test; *P < 0.05.

To evaluate the relationship between CD14^+^CD16^+^ monocyte transmigration across the BBB and HIV-NCI in the context of viral suppression, we isolated PBMCs from PWH on ART. Cells were added to the in vitro BBB model and allowed to transmigrate to CCL2 for 24 hours. Cells that transmigrated were collected. The PBMCs were analyzed by flow cytometry prior to and after transmigration. The gating strategy is presented in Supplemental Figure 1. We quantified the percentage of CD14^+^CD16^+^ monocytes, CD14^+^CD16^–^ monocytes, and CD3^+^ T cells relative to their input that transmigrated across the BBB. The calculations used are shown in Supplemental Figure 2. Among all participants regardless of cognitive status, CD14^+^CD16^+^ monocytes were highly enriched after transmigration to CCL2, comprising a mean of 46.17% (SD = 33.44) of the cells transmigrating across the BBB model (*n* = 48) (Figure 1A). Classical CD14^+^CD16^–^ monocytes had a mean transmigration of 4.21% (SD = 4.16, *n* = 48) (Figure 1A). CD3^+^ T cells had a mean transmigration of 4.78% (SD = 2.75, *n* = 48) (Figure 1A). Compared with CD3^+^ T cells and classical CD14^+^CD16^–^ monocytes, CD14^+^CD16^+^ were not an abundant cell type in the peripheral circulation. However, they were highly enriched after transmigration, with almost half of the total CD14^+^CD16^+^ monocytes transmigrating across the BBB. The percentage of CD14^+^CD16^+^ monocytes that transmigrated was significantly higher than the percentage of classical monocytes and CD3^+^ T cells (*P* < 0.0001 using 1-way ANOVA test with Tukey’s correction for multiple comparisons). This indicates that CD14^+^CD16^+^ monocytes from PWH on ART preferentially transmigrate into the brain compared with classical CD14^+^CD16^–^ monocytes and CD3^+^ T cells.

### PWH on ART with HIV-NCI have higher CCL2-mediated transmigration of CD14^+^CD16^+^ monocytes across the BBB than PWH on ART with normal cognition.

We compared transmigration to CCL2 of PBMCs from study participants with HIV-NCI to PBMCs from those with normal cognition in our ART-treated population. The baseline transmigration of cells across the BBB in response to media with diluent (BSA) differs between participants, due to inherent variability in primary cells. To normalize for these differences, transmigration is expressed as fold-change compared with baseline set to 1 for each independent donor. We found that transmigration of CD14^+^CD16^+^ monocytes across the BBB to CCL2 was significantly higher in those with HIV-NCI compared with those with normal cognition (median fold-change of transmigration to CCL2 over baseline of 2.34 [IQR: 1.86–2.99] and 1.89 [IQR: 1.25–2.48], respectively, *P* < 0.05 using Wilcoxon’s rank sum test, *n* = 48) (Figure 1B). This was not related to the percentage of CD14^+^CD16^+^ monocytes in the periphery, because we did not find any differences in these between PWH on ART with or without HIV-NCI (Supplemental Figure 3). To our knowledge, this is the first report that CD14^+^CD16^+^ monocyte transmigration is associated with HIV-NCI in PWH who are virally suppressed on ART, independent of the major confounders of illicit substance use and HCV coinfection, which are known risks to cognitive impairment and alterations in monocyte biology. There was no difference in the transmigration of classical CD14^+^CD16^–^ monocytes between PWH on ART with HIV-NCI compared with those with normal cognition (median fold-change in transmigration of CCL2 over baseline of 2.30 [IQR: 1.76–2.81] and 1.79 [IQR: 1.30–2.44], respectively, Wilcoxon’s rank sum test, *n* = 48) (Figure 1C). Additionally, we did not find differences in the transmigration of CD3^+^ T cells between those with and without HIV-NCI (median fold-change in transmigration to CCL2 over baseline of 1.21 [IQR: 1.03–1.43] and 1.10 [IQR: 0.98–1.35], respectively, Wilcoxon’s rank sum test, *n* = 48) (Figure 1D).

Having identified an association between CD14^+^CD16^+^ monocyte transmigration and HIV-NCI, we analyzed differences in transmigration examining each individual cognitive domain to characterize further the association between these cells and cognition. The cognitive battery used for HIV-NCI diagnosis generates demographically adjusted domain T scores for motor function, speed of information processing, abstraction and executive function, working memory, memory encoding, memory retrieval, and verbal fluency, as well as a global average of all domains. Using a T score of less than 40 as a threshold for impairment, we compared transmigration of CD14^+^CD16^+^ monocytes between those with and without impairments in each domain, regardless of overall cognitive status. PWH on ART with impairment in working memory had significantly higher transmigration of CD14^+^CD16^+^ monocytes than those without impairment in this domain (*n* = 48, median fold-change in transmigration to CCL2 over baseline of 2.78 and 2.01, respectively, Wilcoxon’s rank sum test, *P* < 0.05) (Figure 1E). We found a similar trend, though not significant, in those with impairments in the domains of abstraction and executive function and of speed of information processing (*n* = 48, Wilcoxon’s rank sum test, *P* = 0.051 and *P* = 0.082, respectively) (Figure 1E). We did not find any significant differences or trends for the remaining cognitive domains when dichotomized by those with and without impairment per domain, and transmigration of any of the cell types quantified. We assessed whether monocyte transmigration could distinguish patterns of cognitive abnormality. This was done by analyzing only the impaired sample (those with HIV-NCI) and assessing correlations between CD14^+^CD16^+^ monocyte and cognitive domain T scores (Supplemental Figure 4). We did not identify any significant associations between transmigration and cognitive scores among only those with HIV-NCI. Thus, these cells do not appear to be driving impairment of specific individual domains. These data underscore that CD14^+^CD16^+^ monocyte transmigration is associated with HIV-NCI but does not appear to drive specific patterns of cognitive deficit in PWH on ART.

### Hypertension and diabetes mellitus are potential effect modifiers of the association between cognition and CD14^+^CD16^+^ monocyte transmigration across the BBB.

**Figure 2:**
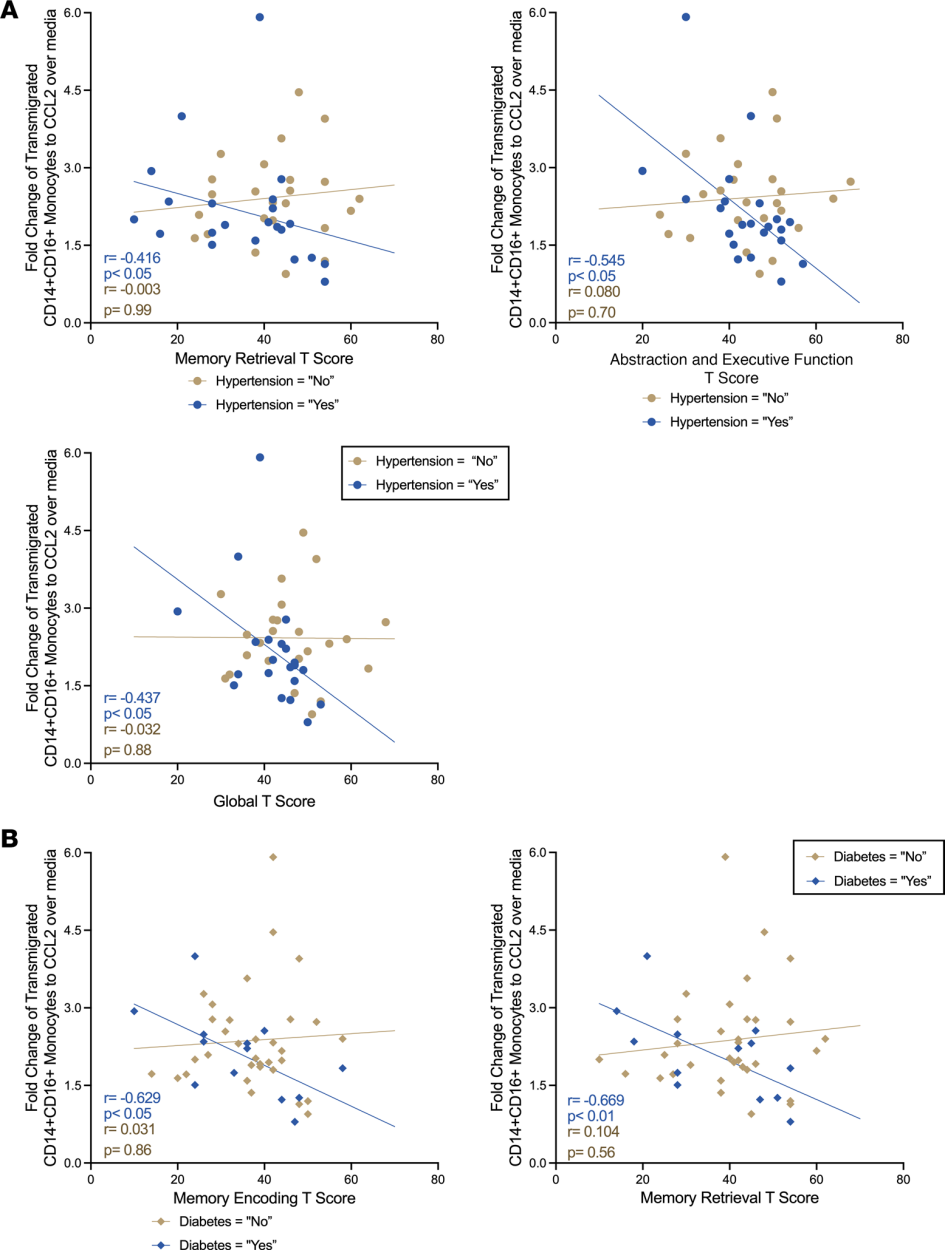
Hypertension and diabetes are effect modifiers on the relationship between cognition and the transmigration of CD14^+^CD16^+^ monocytes across the BBB. (**A**) Associations between fold-change in transmigration of CD14^+^CD16^+^ monocytes and cognitive T scores in the domains in PWH on ART with (*n* = 23) and without (*n* = 25) hypertension. (**B**) Associations between fold-change in transmigration of CD14^+^CD16^+^ monocytes and cognitive T scores in PWH on ART with (*n* = 14) and without (*n* = 34) diabetes. Statistical significance of correlation was determined using Pearson’s correlation test, significance set to *P* < 0.05.

While we excluded major cognitive confounds from our study sample, PWH on ART are affected by other medical comorbidities, many of which are known to increase the risk of cognitive impairment. We examined whether other comorbidities in our study population were correlated with HIV-NCI. We did not find a difference in prevalence of characterized comorbidities in the study population between those with HIV-NCI and those with normal cognition (Table 1). We assessed whether a relationship existed between cognition, transmigration of CD14^+^CD16^+^ monocytes across the BBB, and the comorbidities that had the highest prevalence in our study population to ensure that statistical comparisons could be made. These were hypertension (*n* = 27, 48%), hyperlipidemia (*n* = 28, 50%), diabetes mellitus (DM) (*n* = 16, 29%), and smoking (*n* = 14, 25%) (Table 1). We measured linear correlations between each cognitive domain T score and transmigration of CD14^+^CD16^+^ monocytes dichotomized by comorbidity status (Table 2). For virally suppressed PWH with hypertension, we demonstrated that greater CD14^+^CD16^+^ monocyte transmigration was associated with poorer performance in the domains of speed of information processing (*r* = –0.416, *P* < 0.05), abstraction and executive function (*r* = –0.545, *P* < 0.05), and overall global T scores (*r* = –0.437, *P* < 0.05) (*n* = 23, *P* < 0.05, Pearson’s correlation) (Figure 2A). In contrast, there were no significant correlations between CD14^+^CD16^+^ monocyte transmigration and any of the domain T scores among ART-treated PWH without hypertension (Table 2 and Figure 2A). When we dichotomized by DM, we found higher CD14^+^CD16^+^ monocyte transmigration was associated with poorer T scores in the cognitive domains of memory encoding and memory retrieval among those with DM (*r* = –0.629, *P* < 0.05, and *r* = –0.669, *P* < 0.05, respectively, *n* = 14, Pearson’s correlation) (Table 2 and Figure 2B). We did not find any associations between transmigration and any cognitive domains in those without DM (Table 2 and Figure 2B). Neither hyperlipidemia nor smoking exhibited a similar effect as found with hypertension and DM (Table 2). These findings indicate that hypertension and DM are driving the association between CD14^+^CD16^+^ monocyte transmigration and cognition, suggesting that these comorbidities may be effect modifiers. However, we did not have enough power to measure statistical interactions. Larger studies are needed to determine whether there are significant statistical interactions between hypertension or DM with CD14^+^CD16^+^ monocyte transmigration and cognition.

### ^1^H-MRS–derived neuronal markers of damage do not correlate with cognition or PBMC transmigration.

Participants underwent ^1^H-MRS to characterize the relationship between PBMC transmigration and neurometabolites within our well-characterized cohort of PWH on ART. We analyzed 5 regions of the brain: right and left deep frontal white matter, right and left head of caudate nucleus, and midline frontal gray matter (bilateral anterior cingulate in 1 region of interest). In each region, we quantified *N*-Acetylaspartate (tNAA), total myoinositol (tMI), total choline (tCho), and total glutamine/glutamate (tGlx) and normalized these values with total creatine (tCr). We conducted a principal components factor analysis (PCA, varimax rotation) of the 15 measurements to address correlations between concentrations. Six factors emerged (Table 3) and were defined based on the markers demonstrating the highest factor loadings: *Factor 1*-*tMI*, *Factor 2*-*tCho*, *Factor 3-anterior cingulate*, *Factor 4-tNAA*, *Factor 5-left Glx*, and *Factor 6-right Glx*. The extracted factor scores from the PCA were used in subsequent correlational analyses to examine their relationship with PBMC transmigration and cognitive function. Only 1 significant association emerged after FDR correction using the Benjamini-Hochberg procedure. Greater transmigration of CD14^+^CD16^+^ monocytes was associated with higher *Factor 4-tNAA* scores (Supplemental Table 1). The direction of this association was opposite to expectation, as greater CD14^+^CD16^+^ monocyte transmigration, thought to model a potentially neurotoxic phenomenon, was associated with higher NAA/Cr values that are neuroprotective. We further dichotomized the analysis by cognition, repeating the analyses to measure associations between the factors and the variables in those with normal cognition and those with HIV-NCI (Supplemental Table 2). Dichotomization demonstrated that this association was being driven by those PWH with normal cognition and was lost in those with HIV-NCI (Supplemental Table 2). Because this association did not appear in those with HIV-NCI, it did not seem to have clinical relevance to impairment.

No other significant associations were found between neurometabolites and other clinical variables, including when participants were dichotomized by HIV-NCI status. These data indicate that for this well-defined population of PWH on ART, ratios of neurometabolites, which have served as a measure of CNS dysfunction in several studies that had fewer exclusions, do not correlate to cognition or PBMC transmigration.

## Discussion

HIV-NCI persists despite viral suppression achieved with ART (1). Approximately 15%–50% of PWH on ART manifest HIV-NCI, with some groups reporting a prevalence as high as 55% (1, 2). It is not well understood why some PWH are affected while others are not. Studies identified risk factors that contribute to HIV-NCI (e.g., active viremia, HCV coinfection, and substance use), but mechanisms that contribute to cognitive impairment in PWH on ART are still being characterized. One characteristic of HIV neuropathogenesis is continued chronic low-level inflammation both within and outside the CNS that contributes to neuronal injury (23–25). CD14^+^CD16^+^ monocytes are important mediators of chronic inflammation in PWH, who have a higher proportion of circulating CD14^+^CD16^+^ monocytes than people who are not infected, and this increase persists even with efficacious ART (18, 26, 27). Entry of both HIV-infected and uninfected monocytes into the CNS and their subsequent differentiation into resident macrophages may be key mechanisms of HIV neuropathogenesis, as these cells release neurotoxic viral and host factors that damage neurons and inflammatory cytokines that contribute to additional recruitment of leukocytes into the CNS (7, 28). We previously reported in a study of PWH that included both viremic and ART-suppressed individuals, as well as some with substance use disorder or HCV infection, that those with cognitive impairment had higher transmigration of CD14^+^CD16^+^ monocytes, suggesting that CD14^+^CD16^+^ monocyte transmigration into the CNS serves as a mechanism for HIV-NCI (12). In this current study, we minimized the impact of known confounders of HIV-NCI and demonstrated that, in the context of viral suppression, peripheral blood CD14^+^CD16^+^ monocyte transmigration across the BBB is associated with HIV-NCI. We also examined the relationship of peripheral blood CD14^+^CD16^+^ monocyte transmigration with neuroimaging assessment of neurometabolites in regions of interest known to be impacted by HIV.

In our well-defined cohort of virally suppressed PWH on ART, we demonstrated that transmigration of CD14^+^CD16^+^ monocytes across the BBB to CCL2 was increased in PWH with HIV-NCI compared with those with normal cognition. Our findings suggest that PWH with HIV-NCI have a higher level of ongoing CD14^+^CD16^+^ monocyte entry into the CNS compared with those with normal cognition. Thus, even in the context of viral suppression, CD14^+^CD16^+^ monocyte entry into the CNS may be a mechanism that contributes to cognitive impairment. These findings indicate that reducing CD14^+^CD16^+^ monocyte influx into the CNS is a potential therapeutic approach to reduce HIV-NCI. Other studies of PWH reported associations between circulating monocytes and cognition but without ex vivo assays to show a difference in transmigration across the BBB, and by extension, increased entry into the brain. In a study of virally suppressed women on ART, higher levels of circulating CD14^+^CD16^+^ monocytes correlated with worse cognitive function and were predictive of future impairment (29). In another study of PWH in which 90% were on ART, HIV DNA in CD14^+^CD16^+^ monocytes correlated with cognitive performance (30). These studies and others support a role for CD14^+^CD16^+^ monocytes in the development of HIV-NCI (19).

One feature of our study, in addition to requiring ART adherence with viral suppression, was that all participants were on an ART regimen with a tenofovir-emtricitabine backbone, a common backbone at the time this study was begun. This would reduce differences in monocyte migration that could have arisen if variable ART causes differing impacts on monocytes. ART regimens have been proposed to have variable impacts on HIV-NCI, in part due to differing CNS penetrance and off-target toxicities (31, 32). By restricting study enrollment to PWH on the same ART backbone, we minimized variation that different ART regimens could impart on cognitive function. However, we did not control for the third antiretroviral drug in the ART regimen. We set a threshold of on-study plasma HIV RNA to 1,000 copies/mL, with only 2 study participants demonstrating loads of more than 200 copies/mL. The threshold of 1,000 copies/mL was based on the United Nations Programme on HIV/AIDS definition of suppression in its 90-90-90 targets for patients on ART (33).

We chose to exclude 2 additional major factors that are associated with HIV-NCI, active substance use and HCV coinfection (34–36). HCV infection has been shown to contribute to cognitive impairment, infects monocytes, and alters their patterns of activation (37). Therefore, we eliminated this confounder. Substance use is also associated with cognitive impairment and alterations in immunity (22). We excluded active illicit use of methamphetamines, opioids, cocaine, benzodiazepines, and phencyclidine. Many studies show that substances can affect BBB permeability, monocyte migration into the CNS, and CNS macrophages/microglia (22). Our group reported that PWH on ART and ART-naive PWH who had active substance use had more circulating CD14^+^CD16^+^ monocytes than those without (38). We also showed that dopamine, shown to be elevated in extracellular regions of the CNS by use of illicit substances, increases CD14^+^CD16^+^ monocyte transmigration across the BBB in concert with chemokines (39). These studies demonstrate that substance use can independently lead to increased CD14^+^CD16^+^ monocyte transmigration across the BBB, contributing to cognitive impairment. Exclusion of both HCV coinfection and illicit substance use removed 2 major confounders from our study and their contribution to our measured associations between CD14^+^CD16^+^ monocyte transmigration across the BBB and cognitive function. However, by eliminating these from our study, we were unable to evaluate effects from these factors that are relevant to PWH on ART. Further studies can be designed to determine the effect of these confounders. A limitation of our study is that we were unable to exclude substance use entirely, as we permitted prescribed substances, tobacco, marijuana, and alcohol use. Even so, this is the first study to our knowledge to demonstrate the association of increased peripheral blood CD14^+^CD16^+^ monocyte transmigration across the BBB with HIV-NCI in a well-defined virally suppressed PWH cohort in the absence of HCV infection and illicit substance use.

While we eliminated 2 important factors that can influence monocyte populations and cognition in our participant selection, PWH on ART are at increased risk of other comorbidities, particularly age-related pathologies (40–42). In our study population, substantive numbers of individuals had hypertension, DM, and hyperlipidemia and smoked cigarettes. A large, longitudinal study of HIV-NCI from the CNS HIV Antiretroviral Therapy Effects Research identified that, in addition to ART nonadherence and substance use, presence of severe comorbidity was associated with cognitive decline in PWH on ART (40). Thus, we examined the relationship between comorbidities in our cohort, CD14^+^CD16^+^ monocyte transmigration, and cognition. We dichotomized the study population by presence or absence of the prevalent comorbidities and measured the associations between CD14^+^CD16^+^ monocyte transmigration and cognitive domains. We found significant inverse linear associations between several cognitive domains and CD14^+^CD16^+^ monocyte transmigration in those with hypertension and those with DM, for which higher mature monocyte transmigration correlated with lower T scores in several cognitive domains. These associations were not present in those without hypertension or those without DM. We did not identify any associations between CD14^+^CD16^+^ monocyte transmigration and cognition in those who did or did not smoke or those with or without hyperlipidemia. These findings suggest that in our cohort of virally suppressed PWH without illicit substance use or HCV, hypertension and DM are potential effect modifiers on the association between CD14^+^CD16^+^ monocyte transmigration and cognition.

Hypertension and diabetes increase risk of cognitive impairment in the general population and in PWH (43–48). In a study of PWH and uninfected people that grouped cognitive function into 3 classes (a multidomain impairment class, a learning and recall impairment class, and an unimpaired domain), regardless of HIV status, DM was associated with multidomain impairment, and in PWH, hypertension was associated with multidomain impairment (49). In a cohort of PWH based at our recruitment site, hypertension and longitudinal changes in blood pressure contributed to poorer cognitive test performance, particularly in abstraction/executive function and psychomotor domains (50). The mechanisms by which hypertension and DM contribute to cognitive impairment are likely to be diverse. One proposed way these conditions contribute to CNS dysfunction is through their causal relationship with cerebral small vessel disease (CSVD) (51–53). The pathology of CSVD includes increased expression of adhesion molecules by the BBB that contributes to leukocyte influx into the CNS (51). Our results suggest that, in PWH with hypertension or DM, entry of CD14^+^CD16^+^ monocytes across the BBB into the CNS contributes to CNS dysfunction.

A detailed role of CD14^+^CD16^+^ monocytes in hypertension and diabetes pathogenesis has not been characterized in PWH or in the general population. However, several studies show increased monocyte activation in DM. One study found that monocytes from people with DM have higher inflammasome activation. Another reported upregulation of TLR2 and TLR4 and increased NF-κB activity (54). A separate study that focused on macrophage-derived monocytes from people with DM showed upregulated NLRP3 inflammasome activation, which was mitigated after 2 months of metformin treatment (55). Thus, the inflammatory processes in hypertension and DM in PWH on ART may further promote monocyte activation, making CD14^+^CD16^+^ monocytes even more prone to infiltrate the CNS. In PWH on ART, injury to the BBB by hypertension or DM may promote CD14^+^CD16^+^ monocyte entry, a potential synergistic mechanism contributing to HIV-NCI. It should be noted that both DM and hypertension increase the risk of and can contribute to atherosclerosis (56, 57), and CD14^+^CD16^+^ monocytes are major effectors of HIV-associated atherosclerosis (58). We were unable to assess the contribution of atherosclerosis as it was not assayed in our cohort. Given the interrelationship of hypertension and DM to cardiovascular disease, a limitation in our study is that we were not powered to assess the impact of cardiovascular disease on the association between CD14^+^CD16^+^ monocyte transmigration and cognition. Additionally, we did not have measures of hypertension and DM severity such as hemoglobin A1c determinations or multiple blood pressure readings; thus, we were unable to assess whether these conditions were under adequate medical control.

Another aspect of our study was the use of ^1^H-MRS neuroimaging. We quantified ratios of tNAA/tCr, tCho/tCr, tMI/tCr, and tGlx/tCr in the frontal white matter, caudate, and anterior cingulate. Only 1 association was observed (and in the unexpected direction): CD14^+^CD16^+^ monocyte transmigration and the factor score reflective of tNAA, which, when dichotomized by cognition, only persisted in those with normal cognition. We are unsure of the clinical relevance of this. Our findings that there are no other associations between neurometabolites and cognition or PBMC transmigration, may be due, in part, to the stringency in our inclusion and exclusion criteria. In a previous pilot study, we reported several associations between neurometabolite data and CCR2, the monocyte receptor for CCL2, on CD14^+^CD16^+^ monocytes, but this was for PWH without exclusion of HCV or substance use, and half of the participants who underwent neuroimaging used opioids (59). Several studies reported neurometabolite abnormalities in substance users and correlations between neurometabolite concentration and behavioral changes (60, 61). Given that substance use can cause CNS disturbances, it is possible that opioid use within the previous study population contributed to this finding. Additionally, the range of neurometabolite ratios measured in the current study population is smaller than the range in our prior pilot study, despite using the same method for measurement. The smaller range in neurometabolite ratios for the current study population suggests that the spectrum CNS disturbance is smaller when significant confounders like substance use and HCV are eliminated. Power calculations before implementing the current study were based on our pilot, and the narrower range of neurometabolite ratios may have resulted in our not being powered to identify statistically significant associations between neurometabolites, cognition, and CD14^+^CD16^+^ monocyte transmigration.

Several groups reported that neurometabolite abnormalities indicative of CNS damage persist in PWH despite ART, compared with uninfected individuals. Studies done with cohorts of PWH on ART showed associations between neurometabolite concentrations and serum markers of inflammation (62, 63). For many of these, the contributions of substance use, HCV coinfection, and ART regimen were not well defined, nor was the severity of HIV-NCI. Conclusive associations between specific neurometabolite abnormalities and HIV-NCI or specific cognitive domains in PWH on ART have not been established (64). Considering that for our well-defined cohort of PWH on ART we did not identify associations between neurometabolites and PBMC transmigration or cognition, and that other groups have not established clear associations between neurometabolites and HIV-NCI within other cohorts of PWH on ART using 3 T MRS, these data suggest that 3 T MRS may not be the best imaging modality to identify subtle differences in CNS disturbances between virally suppressed PWH with HIV-NCI and those with normal cognition. Another possibility is that studies to date have been underpowered to detect smaller impacts when aviremic individuals undergo neuroimaging. It is also possible that the severity of other medical confounders plays a role in influencing neurometabolites in manners that are not accounted for in studies of PWH.

The inability to detect associations may be methodologic. An alternative for 3 T MRS is 7 T MRS, which imparts better resolution and can detect more subtle changes than 3 T MRS. One study that used 7 T MRS reported that for a cohort of 45 PWH on ART without substance use, those with HIV-NCI had lower ratios of tNAA/tCr in the frontal white matter and posterior cingulate and reported an association between lower frontal white matter tNAA/Cr with worse performance on measures of executive function, fine motor skill, and psychomotor speed (65). In addition to 7 T MRS, an emerging imaging modality that could be useful for identifying CNS perturbations specific to HIV-NCI in PWH on ART is dynamic contrast-enhanced MRI of region-specific BBB permeability. Future studies focused on identifying associations with BBB permeability in PWH on ART should consider comorbidities as part of the study design.

Overall, our results suggest that PWH with HIV-NCI have ongoing CD14^+^CD16^+^ monocyte entry into the CNS at a higher level than those with normal cognition. These findings indicate that a potential avenue in therapy development for HIV-NCI is to reduce the entry of these cells into the CNS. Two studies performed using EcoHIV-infected mice, which models HIV-NCI in people taking ART, showed that treatment with buprenorphine, an opioid agonist therapy, reduced and prevented cognitive impairment that develops in the mice as a result of EcoHIV infection (66, 67). The studies showed that EcoHIV-infected mice treated with buprenorphine had fewer infiltrating inflammatory monocytes in the brain compared with untreated uninfected mice, indicating that prevention and treatment of cognitive impairment with buprenorphine may have been due, in part, to reduction of inflammatory monocyte entry into the CNS. Thus, blocking of CD14^+^CD16^+^ monocyte transmigration across the BBB may be a successful treatment for HIV-NCI.

One approach to reduce mature monocyte entry into the CNS is to block surface molecules that are essential for the transmigration of CD14^+^CD16^+^ monocytes across the BBB (68, 69). Additionally, the viral reservoir in the CNS can be reseeded by entry of HIV harboring immune cells, and it is unknown whether this reseeding is essential for the maintenance and persistence of the CNS reservoir under ART. Our group showed that among PBMCs that transmigrated across the BBB, there was preferential transmigration of monocytes harboring HIV compared with those without virus (69). Therefore, specifically blocking the entry of CD14^+^CD16^+^ monocytes that carry HIV into the CNS may be an additional therapy strategy. While CCL2 is increased in the CNS of PWH on ART, other chemokines, including CXCL12, are also increased in PWH and can contribute to leukocyte recruitment (70, 71). We previously showed that CD14^+^CD16^+^ monocytes from PWH transmigrated across the BBB to CXCL12 and additional studies are ongoing. Another limitation of this study is that we were unable to measure and correlate CCR2 on CD14^+^CD16^+^ monocytes with cognition.

Our finding that hypertension and DM may be effect modifiers on the association between CD14^+^CD16^+^ monocyte transmigration and cognition indicate that PWH on ART with these comorbidities could be a more vulnerable population that would most benefit from a therapy that limits CD14^+^CD16^+^ monocyte transmigration across the BBB. Therefore, future studies on mechanisms that contribute to HIV-NCI in PWH on ART should consider the effect of comorbidities.

## Methods

### Sex as a biological variable.

Both males and females were recruited into the study. All analyses measured the significance of sex as a covariate; sex was dropped as a covariate when not statistically significant.

### Study participants and procedures.

Participants were recruited to the study at the Icahn School of Medicine at Mount Sinai (ISMMS) using consent documentation and protocols approved by the ISMMS Institutional Review Board (IRB). Approval was also obtained from the IRB at the Albert Einstein College of Medicine, where deidentified blood samples were sent for PBMC isolation and characterization. Eligibility criteria included PWH aged 18 or older, on a stable ART regimen with a tenofovir-emtricitabine backbone for a minimum of 3 months, and whose last plasma viral load in clinical care undetectable. The ART regimen was chosen because it was the most common at the time of study design. Exclusion criteria were evidence of infection with HCV; active use of illicit substances; inability to complete a cognitive test battery (for example, due to blindness or upper extremity amputation); presence of metal implants or other devices that would preclude MRI; and for women, pregnancy.

Once consented, study participants underwent a cognitive test battery that has been utilized by large HIV-NCI consortia such as the National NeuroHIV Tissue Consortium and the CNS HIV Antiretroviral Therapy Effects Research consortium for over 2 decades (72). The battery consists of demographically and educationally normed cognitive tests that are utilized to calculate a global T score and T scores for the following domains: motor, speed of information processing, memory encoding, memory retrieval, working memory, abstraction/executive functioning, and verbal fluency. Study participants were classified as having normal cognition or HIV-NCI according to criteria elaborated in the research nosology created in 2007 (Frascati criteria) (73). Following these criteria, individuals who had a T score below 40 in at least 2 domains were categorized as having HIV-NCI. Medical comorbidities were determined by interview and confirmation by review of the electronic medical record. Comorbidities assessed included hypertension, hyperlipidemia, DM, viral hepatitis, end-stage liver disease (not present in any participant), chronic renal disease, chronic obstructive pulmonary disease, cardiovascular disease, stroke, malignancy, lipodystrophy (not present in any participant), and current smoking of tobacco.

Once cognitive testing was completed, study participants underwent neuroimaging, and on the same day, a blood draw for laboratory analysis and PBMC isolation. Clinical laboratories included CD4^+^ T cell enumeration, plasma viral load determination, and HCV serology (required to be negative). Urine was obtained for toxicology testing for amphetamines, barbiturates, benzodiazepines, opiates, buprenorphine, cocaine, cannabis, methamphetamine, methadone, oxycodone, phencyclidine, and tricyclic antidepressants.

From a total of 62 patients recruited to the study, 56 met study inclusion and exclusion criteria once laboratory testing was conducted. Reasons for exclusion after consent were on-study plasma HIV load greater than 1,000 copies/mL (*n* = 3) and presence of illicit substances in the urine on the date of testing (*n* = 3).

### ^1^H-MRS imaging.

MRI was performed on a Skyra 3 T scanner (Siemens Medical Solutions), utilizing a vendor-provided 20-channel head/neck coil. The acquisition protocol included a sagittal T1-weighted magnetization-prepared rapid gradient-echo (MP-RAGE) acquisition with 224 × 224 × 176 mm^3^ field of view (FOV) and 224 × 224 × 176 imaging matrix. Other acquisition parameters were repetition time (TR) 2,400 ms, echo time (TE) 1.99 ms, inversion time 1,000 ms, and flip angle 8°. For the purposes of chemical shift imaging (CSI) guidance, the MP-RAGE images were reformatted in axial and coronal orientations at 1 mm isotropic resolution on the scanner.Proton spectra were acquired using a vendor-provided multivoxel 2D-CSI point-resolved spectroscopy sequence. The 15 mm–thick axial slice with volume of interest 80 × 60 mm^2^ was placed at the level of the head of the caudate nucleus to cover bilateral frontal white matter, midline frontal gray matter (cingulum), and the head of the caudate nucleus. Other acquisition parameters were FOV = 160 × 160 mm^2^, TE/TR = 30/1,700 ms, matrix = 16 × 16, data points = 1,024, receiver bandwidth = 1,200 Hz, and averages = 3. The collected spectra were fitted using the time domain linear combination software LCModel (version 6.3) (74), and metabolite amplitudes were expressed in arbitrary units. As quality control, voxels with spectral signal-to-noise ratio below 5 and Cramer-Rao lower bound above 25% were discarded from further analysis. Consequently, tNAA, tCho, tCr, myoinositol (tmI), and glutamine and glutamate (Glx) were calculated per voxel, and metabolic amplitude ratios tNAA/tCr, tCho/tCr, tmI/tCr, and Glx/tCr were used as outcomes for analyses.

To produce metabolic maps, the metabolic ratios were overlaid on MP-RAGE using a custom-written software assuming absence of subject motion between the MP-RAGE and CSI acquisitions. Cingulum and left and right caudate nuclei masks were obtained by segmenting MP-RAGE images using freesurfer (75–94), and the 2 frontal white matter masks (95) were transferred to the MP-RAGE using FSL (96).

### Blood processing for PBMC isolation.

Blood samples were collected in EDTA-coated collection tubes and processed within 4 hours of blood draw. PBMCs were isolated by density gradient centrifugation of whole blood overlaid on Ficoll-Paque PLUS (GE Healthcare, now Cytiva). PBMCs were immediately used for antibody staining for flow cytometry and for assessment of transmigration across the BBB model.

### BBB model and transmigration assays.

The in vitro BBB model used for these studies has been extensively characterized (9, 68, 97–101). Primary human astrocytes were obtained from tissue as a part of an approved research protocol at the Albert Einstein College of Medicine, Montefiore Medical Center. Astrocytes and human brain microvascular endothelial cells (BMVECs; Applied Cell Biology Research Institute) were cocultured on opposite sides of a tissue culture insert with a porous membrane (3 μm pores, Corning) and placed in a 24-well tissue culture plate. Astrocytic endfeet processes contact BMVECs through membrane pores, establishing a tight barrier through expression of tight junctions and markers consistent with the human BBB. Prior to transmigration assays, several cocultures were established at the same time to be assayed for permeability. These cocultures were quantified for permeability of the BBB using Evans blue dye coupled to albumin (102). All cocultures were impermeable. For transmigration assays, the BBB cocultures were placed in media containing CCL2 (200 ng/mL, R&D Systems, Bio-Techne) or diluent (0.1% BSA in PBS, Thermo Fisher Scientific). Transmigration of PBMCs from each participant was assessed by adding 4 × 10^5^ PBMCs suspended in media to the top of the BBB coculture, the peripheral side of the BMVECs. PBMCs were allowed to transmigrate in response to CCL2 or diluent for 24 hours. Each condition for transmigration was in quadruplicate. At 24 hours, the BBB cocultures were removed, and the cells that transmigrated were collected from the wells. Transmigrated cells were immediately stained and fixed for flow cytometry.

### Flow cytometry.

Cell surface markers on PBMCs were identified using fluorochrome-coupled antibodies against human CD14 (clone M5E2; BD Pharmingen), human CD16 (clone 3G8; BD Pharmingen), and human CD3 (clone HIT3A; BD Pharmingen) and corresponding isotype-matched control antibodies (clone MOPC-173, BD Pharmingen; clone MOPC-21, BD Pharmingen; and clone G155-178, BD Pharmingen, respectively). Antibodies were titered prior to use to determine optimal concentrations. A total of 200,000 PBMCs were washed once and then kept in the dark on ice for 30 minutes with the appropriate antibodies. PBMCs were then washed and fixed with 1% paraformaldehyde.

Flow cytometry was performed using a BD FACSCanto II flow cytometer or Thermo Fisher Scientific Attune NxT Flow Cytometer and analyzed using FlowJo software (v. 10.7.1, TreeStar). Gating on monocytes was determined using forward- and side-scatter and expression of CD14. Lymphocytes were identified using forward- and side-scatter. Monocyte subset gates were determined using matched isotype control antibodies and fluorescence minus one controls. Classical monocytes were defined as CD14^+^CD16^–^ and intermediate monocytes as CD14^+^CD16^+^. T cells were CD3^+^ lymphocytes.

### Statistics.

Statistical analysis was performed using Prism 9.0 software (GraphPad Software) and STATA/IC 16.1 for Mac. Wilcoxon’s rank sum test, 2-tailed *t* test, or 1-way ANOVA was performed to examine differences between PWH on ART with and without HIV-NCI. Pearson’s correlation test was used to examine associations between continuous variables. Significance was set at *P* < 0.05. Neurometabolite data were reduced into 6 factors using PCA with varimax rotation with Kaiser normalization.

### Study approval.

Written informed consent of all participants was acquired prior to study participation. Participants were recruited to this study using consent documentation and protocols approved by the ISMMS IRB and the Albert Einstein College of Medicine IRB.

### Data availability.

Values for all figures and tables are reported in the Supporting Data Values XLS file.

## Author contributions

JWB and SM conceived and designed the study. VV and RLR performed PBMC transmigration experiments. JAL and SM recruited patients and JAL collected patient samples. JG conducted cognitive assessment. JAL and SM collected clinical data. LF conducted MRI and subsequent data extraction. VV and LHR analyzed the data. VV and JWB wrote the paper. All authors coedited.

## Supplementary Material

Supplemental data

Supporting data values

## Figures and Tables

**Table 1:**
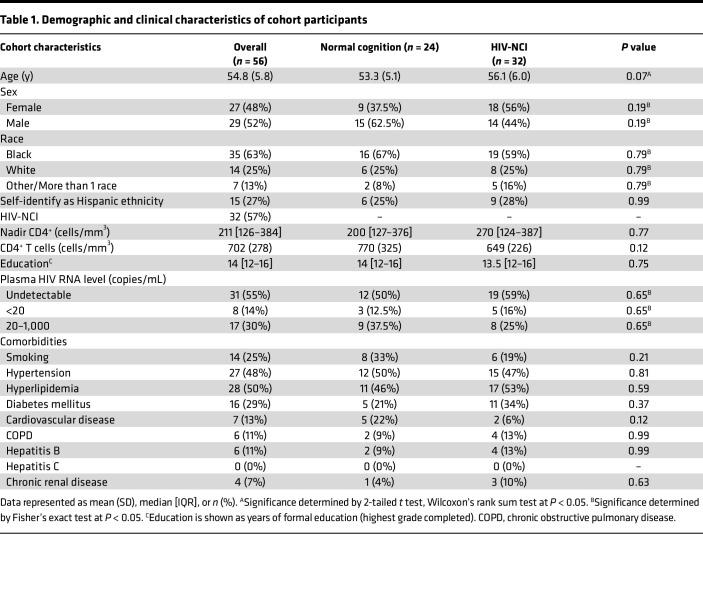
Demographic and clinical characteristics of cohort participants

**Table 2:**
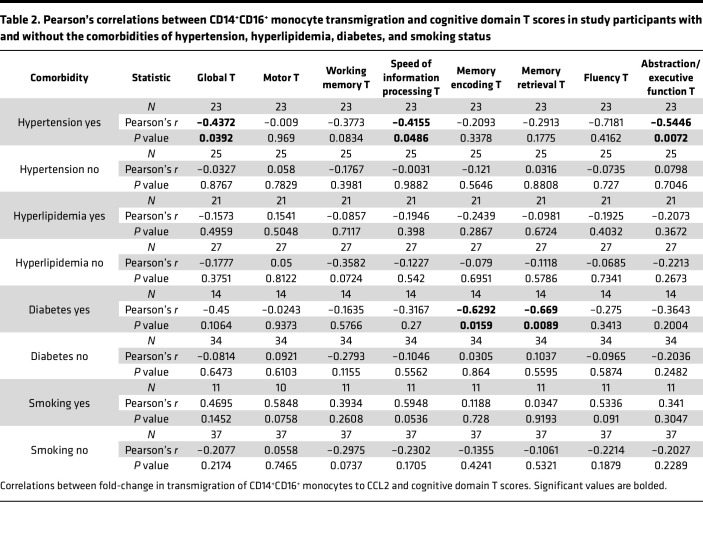
Pearson’s correlations between CD14^+^CD16^+^ monocyte transmigration and cognitive domain T scores in study participants with and without the comorbidities of hypertension, hyperlipidemia, diabetes, and smoking status

**Table 3:**
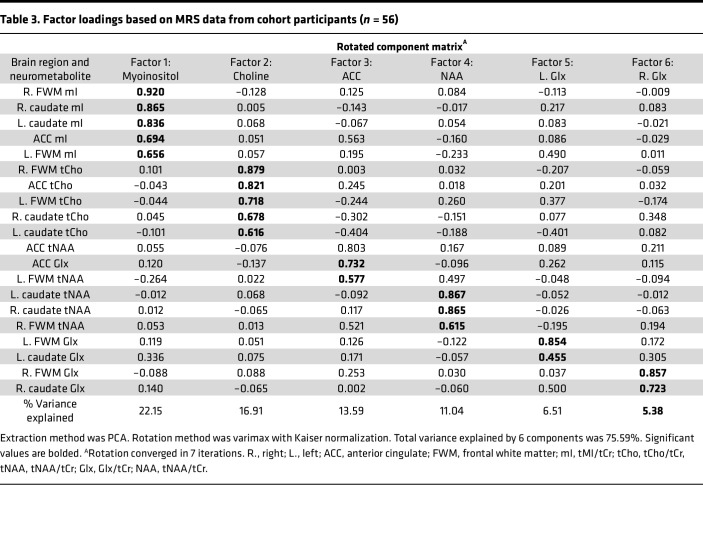
Factor loadings based on MRS data from cohort participants (*n* = 56)

## Version 1 09/10/2024

Electronic publication
