# Supplementary material for: CD14+CD16+ monocyte transmigration across the blood-brain barrier is associated with HIV-NCI despite viral suppression
Source: JCI Insight. 2024 Sep 10;9(17):e179855. doi: 10.1172/jci.insight.179855 (PMC11385088; doi:10.1172/jci.insight.179855)
Supplement: Supplemental data [file jciinsight-9-179855-s036.pdf]

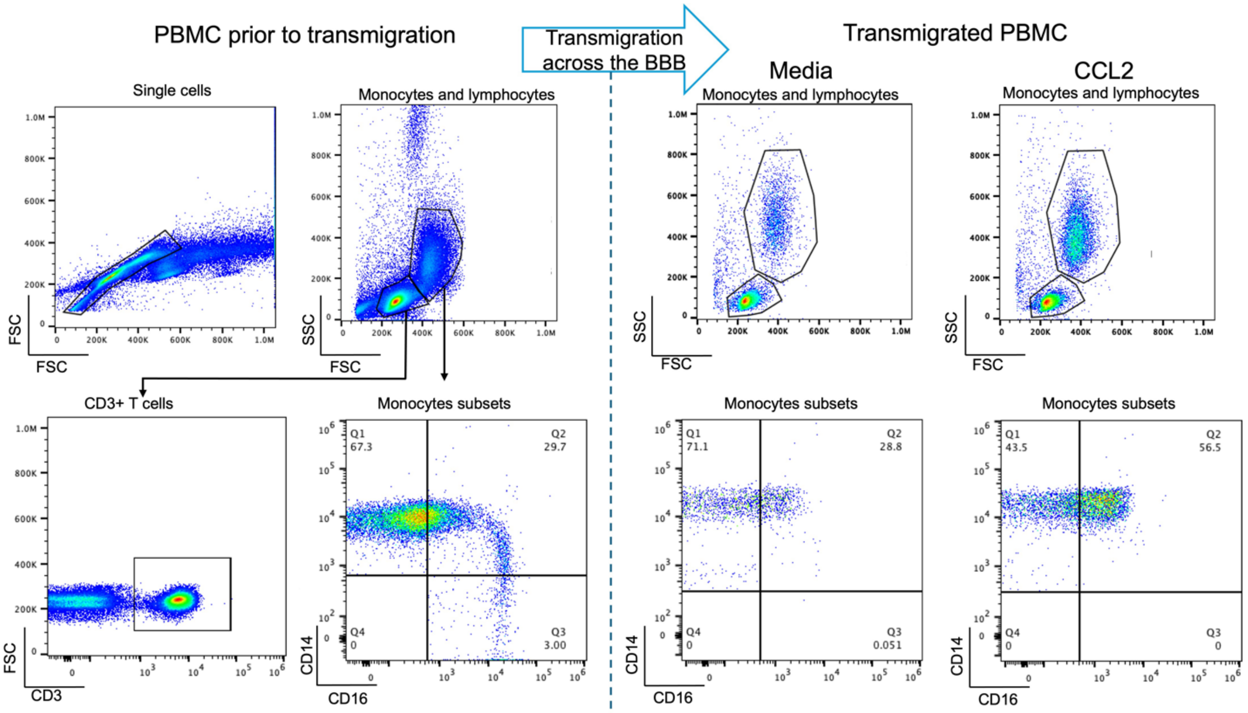

Supplemental Figure 1. Gating strategy. Gating on monocytes was determined using forward- and side-scatter and expression of CD14. Lymphocytes and monocytes were identified using forward- and side-scatter. Classical monocytes were defined as CD14+CD16- and intermediate monocytes as CD14+CD16+. T cells were CD3+ lymphocytes. Gating strategy was consistent for PBMC prior to and after transmigration.

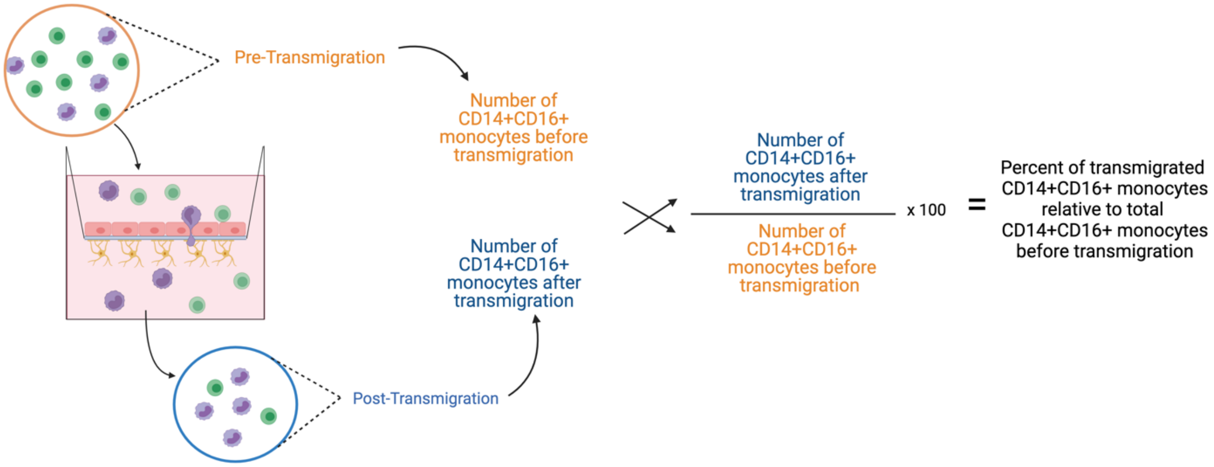

Supplemental Figure 2. Schematic representation of quantification of percent of CD14+CD16+ monocytes that transmigrated across the BBB. In each assay, 400,000 PBMC are added to the top of the tissue culture insert. We quantify the number of CD3+ T cells, CD14+CD16- monocytes and CD14+CD16+ monocytes prior to transmigration and after transmigration with flow cytometry. We divide the number of each subset that transmigrated by the number prior to transmigration to quantify the percent of each subset that cross the barrier. Our criteria for quantification after the assay was at least 400 monocytes after transmigration. Created with BioRender.com

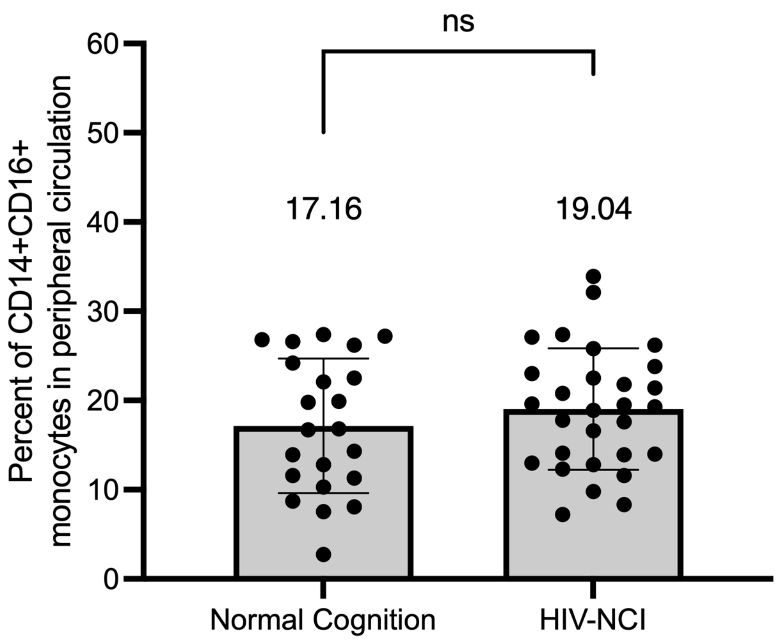

Supplemental Figure 3. There is no difference in the percent of CD14+CD16+ monocyte in peripheral circulation of PWH on ART with and without HIV-NCI. PWH on ART with normal cognition have a mean of 17.16% (SD=7.53) of CD14+CD16+ monocytes in circulation and those with HIV-NCI have a mean of 19.04% (SD=6.79) of CD14+CD16+ monocytes in circulation. Significance set at P<0.05 using two-sided unpaired t-test.

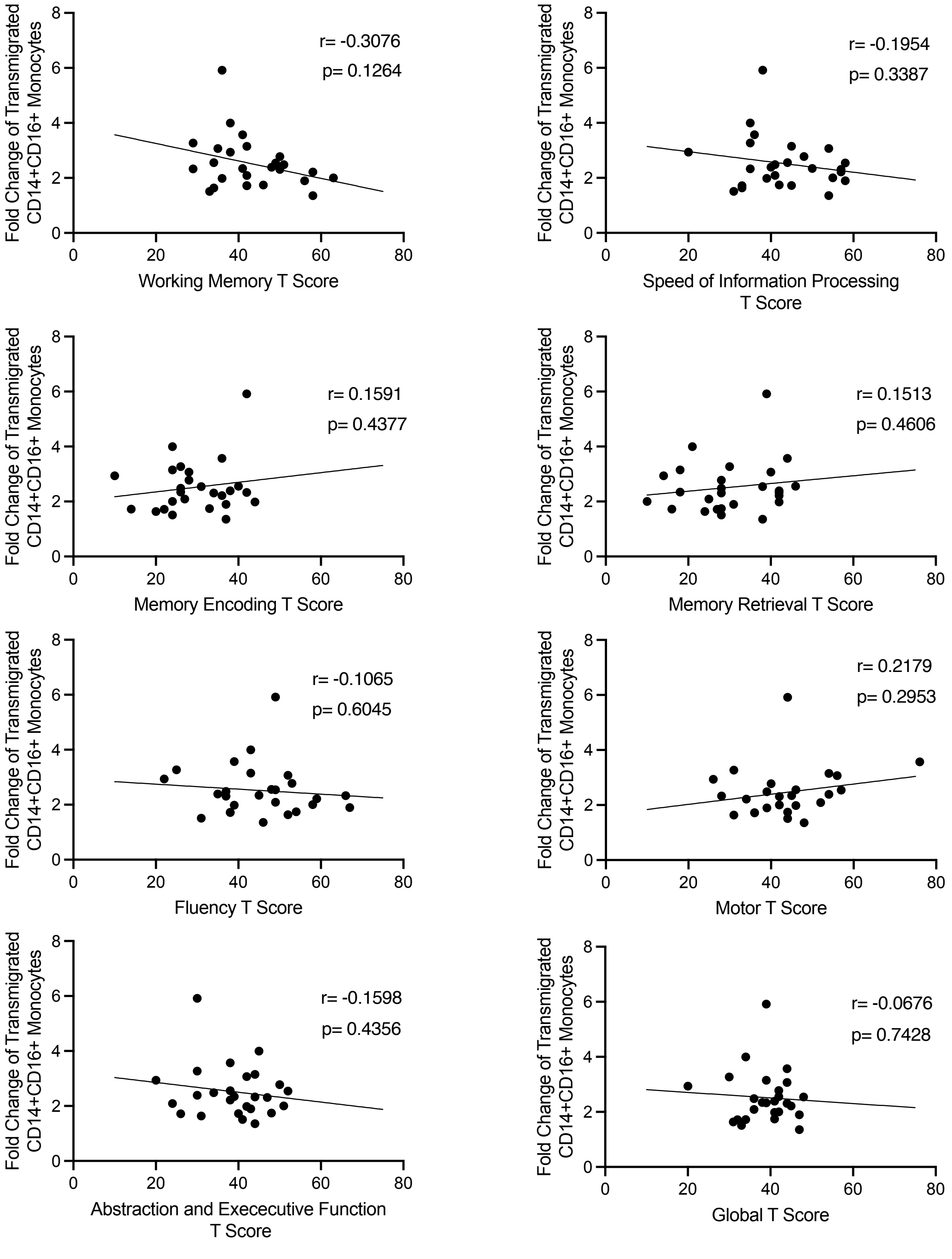

Supplemental Figure 4. CD14+CD16+ monocyte transmigration does not appear to drive impairment of individual cognitive domains.

Pearson correlations between fold change in transmigration of CD14+CD16+ monocytes and cognitive T scores in the domains in PWH with HIV-NCI (n=26).

Supplemental Table 1. Pearson correlation between fold changes in transmigration of CD14+CD16+ monocytes and factor scores (N=48)

Statistic	Factor 1: Myoinositol	Factor 2: Choline	Factor 3: ACC	Factor 4: NAA	Factor 5: L. Glx	Factor 6: R. Glx
r	-.025	.092	-.007	.379**	-.191	-.049
P	.864	.533	.962	.008	.194	.741

** p<0.01 Pearson correlation (2-tailed). L=left; R=right; Glx=XX; NAA=XX; ACC=anterior cingulate

Supplemental Table 2. Pearson correlation between fold changes in transmigration of CD14+CD16+ monocytes and factor scores dichotomized by cognitive status (N=48)

Cognitive Status	Statistic	Factor 1: Myoinositol	Factor 2: Choline	Factor 3: ACC	Factor 4: NAA	Factor 5: L. Glx	Factor 6: R. Glx
Normal	r	-.126	.268	-.191	.539**	-.066	-.279
	P	.578	.227	.395	.010	.771	.209
	N	22	22	22	22	22	22
HIV-NCI	r	.001	.061	.257	.160	-.377	.033
	P	.996	.768	.205	.436	.057	.874
	N	26	26	26	26	26	26

** p<0.01 Pearson correlation (2-tailed). L=left; R=right; Glx=XX; NAA=XX; ACC=anterior cingulate
